# Factors that facilitate diagnosis and brief intervention for risky alcohol use in Brazilian primary health care settings

**DOI:** 10.1186/1940-0640-7-S1-A53

**Published:** 2012-10-09

**Authors:** Erica Cruvinel, Rafaela Lisboa, Michaela Amaral-Sabadini, Telmo Ronzani

**Affiliations:** 1 Department of Social Psychology and Public Health, Federal University of Juiz de Fora, Juiz de Fora, Brazil; 2 Department of Psychology, Federal University of Juiz de Fora, Juiz de Fora, Brazil; 3 Alcohol Unit, Hospital Clinic y Provincial de Barcelona, Barcelona University, Barcelona, Spain

## 

Strategies of diagnosis and brief intervention (SDBI) have been presented as means of preventing ongoing risky alcohol use among Brazilian primary-care patients. To identify factors that facilitate the implementation of SDBI measures in primary care, we conducted a qualitative analysis among 10 community health workers from Zona da Mata, Minas Gerais, Brazil. Participants were divided into two groups: successful outliers (those who regularly administered the Alcohol Use Disorders Identification Test [AUDIT] to patients) and unsuccessful outliers (those who did not regularly administer the AUDIT). We used semistructured interviews and thematic content analysis to identify personal SDBI facilitators (good relationship with the community, satisfaction and commitment to work, feeling prepared to visit patients, feeling comfortable talking about alcohol) and organizational SDBI facilitators (planning, activities organization, and involvement of a multidisciplinary team). We concluded that the factors facilitating successful SDBI implementation were related to both participants’ personal characteristics and organizational factors.

